# Antibody Engineering for Optimized Immunotherapy in Alzheimer's Disease

**DOI:** 10.3389/fnins.2018.00254

**Published:** 2018-04-23

**Authors:** Isabelle L. Sumner, Ross A. Edwards, Ayodeji A. Asuni, Jessica L. Teeling

**Affiliations:** ^1^Biological Sciences, University of Southampton, Southampton, United Kingdom; ^2^Biologics, H. Lundbeck A/S, Copenhagen, Denmark

**Keywords:** amyloid, antibody engineering, immunotherapy, Alzheimers disease, neuroinflammation

## Abstract

There are nearly 50 million people with Alzheimer's disease (AD) worldwide and currently no disease modifying treatment is available. AD is characterized by deposits of Amyloid-β (Aβ), neurofibrillary tangles, and neuroinflammation, and several drug discovery programmes studies have focussed on Aβ as therapeutic target. Active immunization and passive immunization against Aβ leads to the clearance of deposits in humans and transgenic mice expressing human Aβ but have failed to improve memory loss. This review will discuss the possible explanations for the lack of efficacy of Aβ immunotherapy, including the role of a pro-inflammatory response and subsequent vascular side effects, the binding site of therapeutic antibodies and the timing of the treatment. We further discuss how antibodies can be engineered for improved efficacy.

## Introduction

Alzheimer's Disease (AD) is the most common cause of dementia worldwide, characterized by variable mood changes, difficulty carrying out daily tasks, confusion, and progressive memory loss. An estimated 0.5 million people in the UK have Alzheimer's disease currently, and majority of these subjects 5 are age 65 or older. Which would suggest that as this population ages, the incidence of dementia will increase significantly. In fact, the incidence of AD worldwide is projected to triple by 2050 (https://www.alzheimers.org.uk/info/20007/types_of_dementia/2/alzheimers_disease). Additional risk factors for AD which are beyond the scope of this review, include diabetes, high blood pressure, obesity, smoking, depression, as well as physical, and cognitive inactivity. Crucially, most of these are modifiable which gives hope to effort to reduce the incidences of AD.

The current standard of care, such as donepezil (Aricept®), galantamine (Reminyl® or Razadyne®), and rivastigmine (Exelon®) only alleviate the symptoms by increasing synaptic function and presently there are no approved therapies that can halt the progression of this dilapidating disease. AD is estimated to affect millions of people worldwide (Cynis et al., 2016) and numbers are predicted to increase as our population ages; therefore it is vital to find treatments to stop this disease, or delay the time hospitalization and institutionalization of the patients. The success rate of approving novel drugs is very low; with only 9.6% of candidates that enter clinical trials gaining FDA approval (www.bio.org; Cummings et al., 2017); the outlook for Alzheimer's drugs is even bleaker, with an approval rate of only 0.4% between 2002 and 2012; one of the poorest success rates of any disease (Cummings et al., 2014). There is good evidence that some lifestyle changes could alter the incidence of disease. One such change could be improvement of sleep quality. There is increasing evidence that poor sleep leads to higher levels of Aβ in the brain, and in turn aberrant Aβ levels further interferes with sleep and by extension memory consolidation (Diekelmann et al., 2009; Carvalho et al., 2018). This would suggest that targeting sleep represents a future avenue for treating AD. However, discussion on this topic are beyond the scope of this review. Over the last decade, amyloid beta targeting immunotherapy has been at the fore of drug discovery for AD. Some progress has been made as have missteps. In this review we will describe the past, present and future directions of amyloid beta targeting immunotherapy and its potential as a disease modifying therapy for AD.

## APP processing and Aβ accumulation

**Figure 1:**
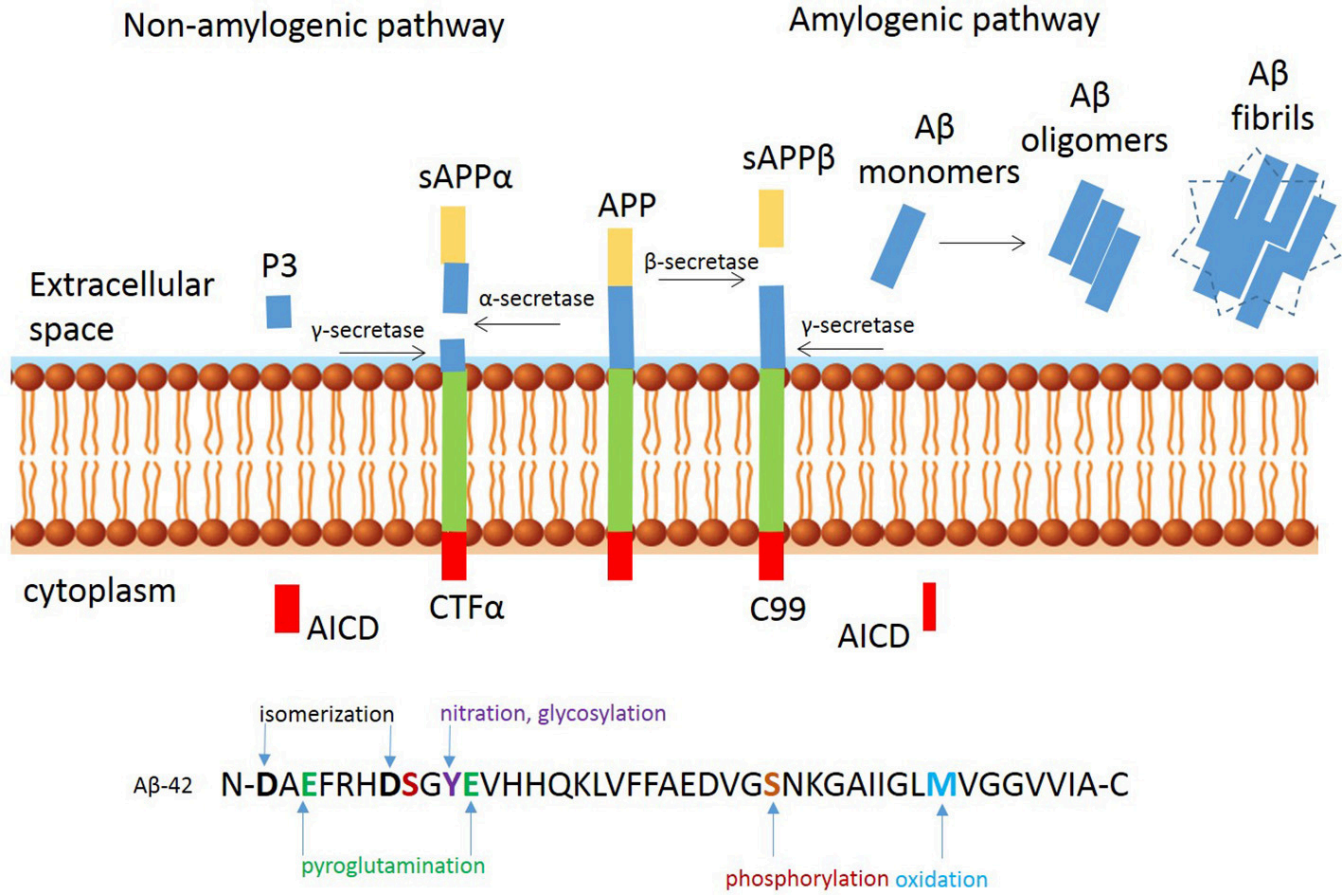
Proteolytic processing of amyloid precursor protein. Proteolytic processing of APP within the non-amyloidogenic **(left)** and amyloidogenic **(right)** pathways and post translational modifications of Aβ-42 peptide at the N-and C-terminus.

Aβ peptide has been the therapeutic target of a number of high profile drug discovery programmes, including both active and passive immunotherapy for AD, based on the “Amyloid Hypothesis” [see for details review hardy and Selkoe (Selkoe and Hardy, 2016)]. Aβ is produced from the cleavage of the amyloid precursor protein (APP) by cysteine proteases and secretase activity (Perez-Garmendia and Gevorkian, 2013; Perez-Garmendia et al., 2014). APP is a type-I membrane protein with its amino N-terminus in the lumen/extracellular space and its carboxyl C-terminus in the cytosol, which can be proteolytically processed by three secretases called α-, β-, and γ-secretase (3). The process is summarized in Figure 1. The non-amyloidogenic pathway is initiated by α-secretase releasing sAPPα into the intracellular space. The resulting CTF-α fragment is cleaved by γ-secretase in the intermembrane space resulting in the AICD and p3 fragments, which do not form plaques. The amyloidogenic pathway is initiated by β-secretase and results in release of sAPPβ and generation of a carboxy-terminal fragment (C99), which is cleaved by γ-secretase and generates monomeric Aβ species including Aβ1-38, Aβ1-40, and Aβ1-42; the latter fragment is prone to aggregate and forms oligomers and fibrils. APP processing occurs naturally in the process of aging, and the resulting peptides are cleared from the brain through bulk flow along the perivascular pathway (Morris et al., 2014). Increasing evidence supports that excessive production or lack of clearance can result aberrant extracellular and intraneuronal accumulation and deposition of Aβ, followed by dysfunction of synapses and eventual loss of neurons. This imbalance between the clearance and production of Aβ peptides forms a critical part of the “Amyloid Hypothesis” (Selkoe and Hardy, 2016). The extracellular plaques exist in two main forms: neuritic or diffuse plaques. Neuritic plaques have a crystalline structure and are able to bind the dye Congo red. These congophilic extracellular deposits of Aβ are associated with degenerative neural structures (dendritic neurites) and are the harbinger for neuroinflammation, as evidenced by an abundance of microglia in the core of the plaque and astrocytes at the periphery (Perl, 2010). Diffuse plaques are not able to bind Congo red and do not disrupt the neuropil. These diffuse plaques are seen in aged persons and are generally not associated with dementia (Morris et al., 1996). Different sizes and self-assembly species of Aβ peptide have been described, with Aβ1-42 typically considered to be the type most prone to aggregate, but there is good evidence that Aβ1-43 for example as well as other modified Aβ species, are more prone to aggregation and more neurotoxic (Saito et al., 2011). The monomers of Aβ peptides can form oligomers, which become larger protofibrils, leading to fibril and plaque formation. It is still not entirely clear how long oligomers of these peptides need to be present in the brains of subjects before they deposit as the insoluble amyloid plaques, but increasing evidence suggests that neuronal toxicity depends on the molecular composition, rather than the amount of these peptides (Piccini et al., 2005).

In addition to most commonly described Aβ1-40 and Aβ1-42 peptides starting with an aspartate at position 1, several other Aβ peptides species have been identified in AD brains (Bayer and Wirths, 2014). The heterogeneous pool of peptides is a result of post-translational modifications, including isomerization and racemization of aspartates, cyclization of N-terminal glutamates, oxidation of methionine and abundant N- and C-terminal truncations. See Figure 1 for a schematic overview of the most common modifications. C-terminal truncations may occur in the presence of metalloproteinases (Cabrera et al., 2018) resulting in a more soluble and less aggregation prone peptides. Examples of C-truncated peptides include Aβ1-38 and Aβ1-37, which are predominantly observed in the vasculature (Moro et al., 2012; Reinert et al., 2016). Oxidation and nitration, are likely induced by the inflammatory milieu (Kummer and Heneka, 2014) and N-terminal truncations arise from the combined actions of Aβ-degrading enzymes, such as neprylisin, insulin degrading-, and endothelin converting-enzymes which are commonly found in human AD, but not in transgenic mice (Kalback et al., 2002; Schieb et al., 2011). An example of an N-terminal modified peptide is Aβ modified by pyroglutamate (AβpE3 or AβpE11) (Jawhar et al., 2011). These peptides have been found in early stages of AD, prior to clinical symptoms. Pyroglutamation occurs after dehydration of glutamate residues, which results in the loss of a negative charge that increases the β-sheet content, aggregation propensity, hydrophobicity, and resistance to enzymatic degradation of the Aβ fragments (He and Barrow, 1999). It mainly localizes in the core of amyloid plaques, suggesting a possible role in inducing and facilitating Aβ oligomerization and accumulation (Dammers et al., 2017). One of the first Aβ peptides reported was the N-terminal truncated Aβ4-42 (Kummer and Heneka, 2014), which is abundant in the hippocampus and cortex of sporadic AD, familiar AD and vascular dementia (Lewis et al., 2006; Portelius et al., 2010). The relevance for this modification in neuropathology is emphasized in transgenic mice expressing Aβ4-42 that develop CA1 neuronal loss and age-related spatial memory deficits without plaque formation (Bouter et al., 2013). These peptides have become a desirable therapeutic target for AD (Perez-Garmendia and Gevorkian, 2013; Cynis et al., 2016). NT4X-167, an experimental antibody that recognizes phenylalanine at position four of Aβ4-x on monomeric and oligomeric Aβ4–x peptides rescued Aβ4-42 induced neuron death *in vitro* and *in vivo* (Antonios et al., 2013, 2015). Supportive immunohistological studies using NT4X-167 detected only a minor fraction of plaques in brain from sporadic and familial AD patients and preferentially reacts with intraneuronal Aβ, rather than plaques in young 5xFAD mice. However, this antibody also recognizes AβpE3–x peptides and is therefore not suitable for accurate measurement of the abundance and distribution of Aβ4–x peptides. Polyclonal antibodies that selectively bind the six-amino acid peptide (FRHDSG) corresponding to residues 4–9 of the Aβ peptide sequence have shown that the distribution of Aβ4–x peptides is restricted largely to amyloid plaque cores and as Aβ deposits found around cerebral blood vessels termed cerebral amyloid angiopathy (CAA) whereas diffuse amyloid deposits were negative for N-truncated peptides. These observations were made in brain sections of both patients with sporadic AD and at very early time points in two AD mouse models (Casas et al., 2004; Wirths et al., 2017). These observations confirm mass spectrometry studies from 30 years ago, indicating a high percentage of N-terminal truncated peptides in plaques, while full length amyloid peptides are more abundant in the vasculature (Masters et al., 1985; Miller et al., 1993). In the same vain, others have shown that a plaque binding antibody targeting a modified AβpE3-42, showed robust clearance of pre-existing plaque (Demattos et al., 2012), and both passive and active immunization with AβpE3 reactive antibodies have also shown to be success for clearance of plaque in APPswe/PS1ΔE9 mice (Frost et al., 2015).

Although AD pathology has been mostly linked to insoluble, aggregated amyloid, soluble species of Aβ also contribute to neuronal dysfunction. Soluble oligomeric species of Aβ have been demonstrated to impair hippocampal LTP (Lei et al., 2016). These soluble species of Aβ can bind to neuronal receptors expressed on synapses, such as N-methyl-D-aspartate receptor (NMDA-R), disrupt glutamatergic/GABAergic balance, and lead to neuronal dysfunction or eventual death. Given the observation that metalloproteinases may be implicated in generating C-terminal modifications, it is tempting to speculate a role for inflammation in increasing the levels of soluble amyloid. Studies using NMDA-R antagonists, such as memantine, to prevent the disruption of synaptic plasticity by soluble Aβ (Hu et al., 2009; Freir et al., 2011), provide additional supportive evidence for the benefits of targeting these peptides as a therapy. It has also been suggested that conformational changes of the receptor, possibly due to increased oxidative stress, rather than flow of ions through the channel, is required for Aβ-mediated synaptic depression (Kessels et al., 2013).

## Anti-Aβ immunotherapy

Targeting Aβ by active or passive vaccination has received much interest from both the pharmaceutical industry and academia in the past two decades. Active vaccination is defined by introducing an exogenous substance to stimulate the immune system to mount a response. The type of immune response can be influenced by certain adjuvants to promote a humoral, or antibody response. Passive vaccination involves the introduction of antibodies directly into an animal or person to produce a benefit similar to that of active vaccination. Numerous passive and active immunotherapeutic approaches were developed for AD, summarized by Brody and Holtzman (2008). In 1999, Dale Schenk et al. published a landmark paper on active Aβ vaccination to prevent and treat amyloid load and cognitive decline in experimental AD models. The active vaccine (AN-1792) was tested in phase 2a clinical trials, but when 6% of patients treated developed encephalitis the development of AN-1792 was terminated, although follow-up assessment of treated patients continued (Nicoll et al., 2003; Maarouf et al., 2010). The learnings from these initial efforts at active vaccination continue are still being absorbed by the field, and additional active vaccination programs have commenced in the last few years with development of CAD106 being the most advanced (NCT02565511).

**Figure 2:**
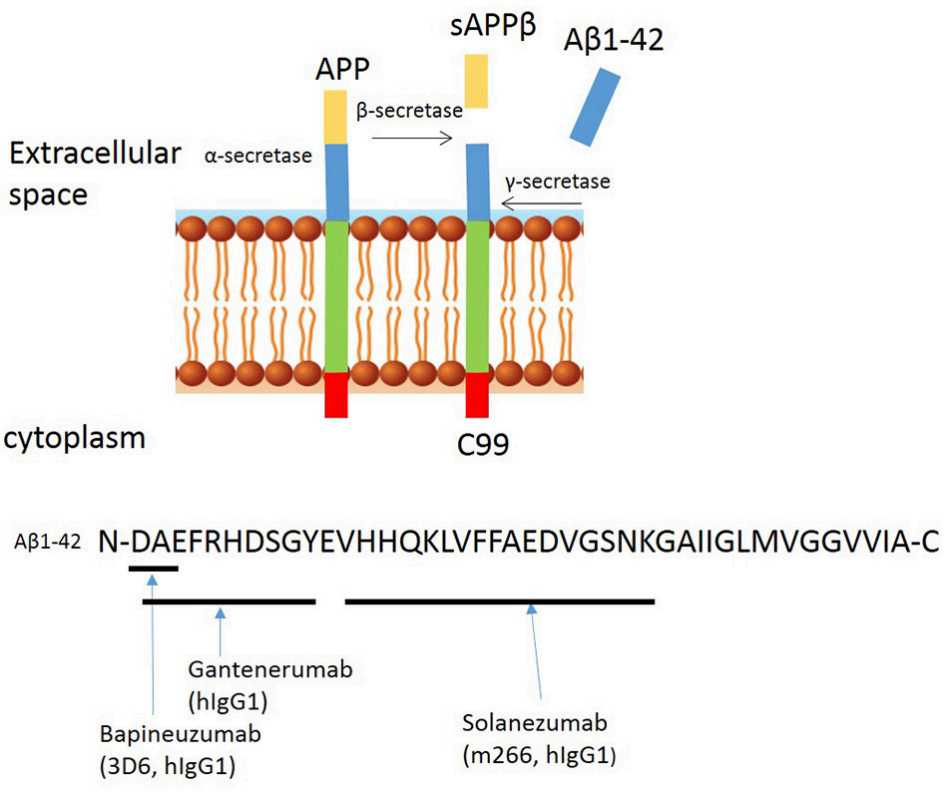
Proteolytic processing of amyloid precursor protein and binding sites of anti-amyloid β antibodies to Aβ peptide. Proteolytic processing of APP within the amyloidogenic pathway and binding sites of Bapineuzumab, Gantenerumab, and Solanuzumab.

Passive immunization approaches using monoclonal antibodies specifically targeting different epitopes of the Aβ peptide have received increasing interest. These antibodies include the widely described Bapineuzumab, Gantenerumab, and Solanezumab, which were all tested in phase 3 clinical trials aiming to become the first disease-modifying therapy for AD. The binding sites of these antibodies are summarized in Figure 2.

Bapineuzumab (AAB-001) is a humanized IgG1 anti-Aβ antibody, derived from the murine monoclonal antibody 3D6 (IgG2b), originally developed by Elan. Bapineuzumab binds Aβ at the N-terminal residues in a monomeric helical conformation (Miles et al., 2013), explaining its selective binding to fibrillar and soluble Aβ species but not truncated peptides (Feinberg et al., 2014). Preclinical studies show that systemic administration of 3D6 lowers plaque load in 9 month old PDAPP mice and levels persist for nearly 1 month in disease-affected brain regions of transgenic PDAPP mice, suggesting penetration across the BBB (Bard et al., 2012). In phase 1 trials, the safety, tolerability, and pharmacokinetics of Bapineuzumab was assessed in patients with mild to moderate AD. Three of the patients receiving the highest dose (5 mg/kg) developed abnormalities as assessed by magnetic resonance imaging (MRI), consistent with vasogenic edema, but it was concluded that the treatment appeared overall safe and well-tolerated. In phase 2 trials (NCT00606476), Bapineuzumab treatment resulted in a greater reduction of amyloid as observed by positron-emission tomographic amyloid imaging using Pittsburgh compound B (PIB-PET) (Rinne et al., 2010). However, there were side effects, including vasogenic edemas (termed amyloid-related imaging abnormalities, ARIA-E) and higher frequencies of micro-hemorrhages compared to control groups (Salloway et al., 2009). ARIA-E, is an increase in extracellular volume due to an increase in the permeability of the BBB to serum proteins. ARIA-E shares characteristics with CAA as both occur in the leptomeninges, gray and white matter (Sperling et al., 2011), and both have an association with the ApoE4 mutation, which increases the risk of developing ARIA-E (Strittmatter et al., 1993); based on these observations, the highest dose (5 mg/kg) was abandoned. Bapineuzumab was tested in phase 3 clinical trials (NCT00575055 and NCT00574132). Both studies were terminated early finding no difference in cognitive decline between the Bapineuzumab and placebo groups (Vandenberghe et al., 2016). Biomarker analyses indicated that Bapineuzumab engaged its target but had no benefit.

Solanezumab (also known as LY2062430), is a humanized IgG1 version of the murine antibody m266, developed by Eli Lilly and Company. This monoclonal antibody targets residues 13–28 (see Figure 1), a mid-terminal epitope of Aβ (Crespi et al., 2015). Solanezumab is unable to bind fibrillary Aβ but instead binds to circulating and soluble Aβ species (Hefti et al., 2013). Preclinical studies showed that systemic administration of m266 removes soluble species of Aβ that are directly toxic to synaptic function, without affecting amyloid plaques (Dodart et al., 2002). Subsequent, phase 1 and 2 clinical trials demonstrated a good safety profile and encouraging indications on both cerebrospinal and plasma biomarkers, including levels of Aβ40, Aβ42, and plasma pyro-Glu Aβ (Imbimbo et al., 2012). The phase 3 trials of this drug (EXPEDITION 1 and EXPEDITION 2) showed no significant improvement in primary outcomes, despite only 0.9% ARIAs with Solanezumab and 0.4% with placebo (Doody et al., 2014). Another phase 3 trial (EXPEDITION 3) was carried out, involving patients with mild AD but has also since been terminated due to failure to reach primary endpoints and no significant difference between Solanezumab and placebo.

Gantenerumab is fully human IgG1 antibody, which binds to a conformational epitope of Aβ consisting both N terminal and central amino acids (Figure 1). It prefers binding to the fibrillary forms of the protein (Novakovic et al., 2013). Preclinical studies reveal that Gantenerumab recruits microglia to reduce amyloid load through antibody mediated phagocytosis, and prevents new plaque formation. This antibody does not alter systemic levels of Aβ which suggested that clearance of soluble Aβ is undisturbed (Bohrmann et al., 2012). In the phase 3 multi-center, randomized, double-blind, placebo controlled trial SCarlet RoAD (Scheltens, 2015). Gantenerumab showed target engagement, resulting in clearance of plaques, and reduced levels of phosphorylated tau in the spinal cord fluid, but the trial was abandoned in December 2014, likely as a result of ARIAs and abandonment of the top dose (Piazza and Winblad, 2016). Unperturbed, two new phase 1 trials of higher doses of Gantenerumab have been announced in 2016, and in 2017 two new phase 3 trials in prodromal AD were announced.

### What have we learned from these trials?

The phase 3 clinical trial of passive immunotherapy targeting amyloid were disappointing given the initial success in experimental models, but have increased our basic understanding of AD. Numerous hypotheses have been generated to explain the failed primary endpoints. These include, but are not limited to (a) the role of antibody effector function and promoting inflammation through Fc receptors, (b) the optimal timing of the therapy, and (c) target engagement and antibody specificity.

#### Antibody effector function

Experimental models of AD and observations from clinical trials have provided evidence that IgG Fcγ Receptors (FcγR) are largely responsible for amyloid clearance following immunotherapy, mediating activation of microglia, and antibody-mediated phagocytosis, but these effects are also associated with increased inflammation and vascular side effects (Wilcock and Colton, 2009). Human IgG1 and mouse IgG2a are the preferred isotype to promote amyloid clearance, due to their higher affinity to activating FcγRs as compared to other IgG subclasses, resulting in more effective phagocytosis and pro-inflammatory cytokine production by the effector cell (Bruhns, 2012). Both microglial activation and micro-hemorrhage are prevented when anti-Aβ antibodies are deglycosylated, confirming the critical role of FcγRs in vascular side effects, at least for antibodies binding the N-terminal epitope of Aβ (Wilcock et al., 2006; Freeman et al., 2012). To study the role of antibody effector function further, we directly compared three antibodies in the same pre-clinical model, with the same IgG2a effector function, for their ability to clear plaques and induce inflammation in the brain (Fuller et al., 2015). The results suggest that the ability of an antibody to safely clear plaques in experimental models is dependent on both the epitope and affinity of the antibody, rather than antibody effector function only. The effector function of IgG can be modified by antibody engineering and this approach has already resulted in a number of second generation amyloid-specific antibodies. Crenezumab (MABT5102A, RG7412) is a humanized IgG4 antibody that can bind to multiple forms of Aβ, and is selective for the mid terminus (Crespi et al., 2015). Using an IgG4 subclass reduces affinity for activating FcγRs and therefore a reduced microglial activation and reduced vascular damage (Bruhns, 2012). Indeed, doses up to 15 mg/kg are well-tolerated in experimental models and AD patients (Adolfsson et al., 2012). Despite these modifications, the ABBY phase 2 trial of Crenezumab, failed to reach its primary endpoints of an improvement in ADAS-Cog12 and CDR-SB score (Soejitno et al., 2015). Possible reasons for lack of efficacy could have been due to the inclusion of patients with moderate AD rather than mild AD, who showed improved cognition. The ongoing CREAD study was designed to test the efficacy of Crenezumab in patients with mild AD, with an estimated study completion date of July 2021. Another antibody, GSK933776, is a humanized IgG1 monoclonal antibody directed against the N-terminus of the Aβ peptide (aa1–5) (Novakovic et al., 2013). The Fc part of this monoclonal antibody is engineered by introducing alanine at position 235 and 237 in the constant region of the heavy chain. Phase 1 trials have been completed (Andreasen et al., 2015) and total levels of circulating Aβ increased and levels of free Aβ in the CSF decreased implying that this Fc-engineered anti-Aβ mAb engaged its target in plasma and CSF without causing brain ARIA-E/H in patients with mild AD or mild cognitive impairment (Leyhe et al., 2014; Andreasen et al., 2015). A phase 2, proof-of-concept study (NCT01342926), evaluated the effects of GSK933776 in patients with Dry Age related Macular degeneration (AMD), but failed to meet its primary endpoint. As part of this study, MRI's were performed and 2/15 participants showed asymptomatic adverse events; a cerebral hemorrhage and a cerebral infarct, both in the 15 mg/kg group. Another antibody, AAB-003, is a humanized Fc engineered version of Bapineuzumab, co-developed by Janssen and Pfizer. To achieve this Fc effector function reduction, three amino acid mutation in the lower hinge region of Bapineuzumab were introduced, reducing the affinity to FcγR and reduced binding to complement C1q (unpublished Janssen data). The AAB-003 was tested in a first in human study and was safe and well-tolerated up to 8 mg/kg in mild to moderate AD (Delnomdedieu et al., 2016). Asymptomatic and resolvable ARIA-E was observed after the first or second infusion of AAB-003, but the dose at which ARIA-E was observed was considerably higher compared to Bapineuzumab (1 mg/kg), a finding that supports the hypothesis that reducing Fc-receptor effector function may reduce the risk of ARIA associated with monoclonal antibodies targeting aggregated cerebral amyloid. Furthermore, another antibody, MEDI 1814, is a high-affinity, fully human IgG1λ monoclonal antibody directed to the C-terminus of Aβ42. This monoclonal antibody is designed to target monomeric Aβ42 and not Aβ40. Its effector function has been reduced with a triple mutation in its Fc tail. In rats and monkeys, MEDI1814 increased total Aβ42 and decreased free Aβ42 in the CSF, without changing Aβ40 levels and no serious adverse events have been reported. None of the participants on drug in phase I had signs of either ARIA-H or ARIA-E (https://clinicaltrials.gov/ct2/show/NCT02036645). This antibody is being developed jointly by AstraZeneca and Eli Lilly.

#### Timing of therapy

The disappointing results of Aβ immunotherapy may be due to the doses of Bapineuzumab used in the studies, as well as the disease state combined with accurate diagnosis of AD. It is estimated that ~20% of patients in the Bapineuzumab trial had dementia, but not as a result of AD (Salloway et al., 2014). The gold standard method for diagnosing AD is histopathology on post-mortem tissue, but amyloid tracers, such as 18F-FDG and 11C-PiB PET have been developed and are now widely used, allowing analysis of plaque deposition during disease course and enrolling patients at earlier stage disease. A more diverse set of diagnostics can identify patients before amyloid plaques or tau tangles appear, which includes the use of CSF and plasma biomarkers that could be used to stratify patients for clinical trials. Examples of biomarkers include CSF levels of amyloid and tau or the more recent described inflammatory markers, including proteins of the complement pathway (Sattlecker et al., 2016) and neurofilament light chain (Lista et al., 2017). Identification of accurate and suitable fluid biomarkers requires more research, using longitudinal studies, which could be accelerated if data and biological samples from past and ongoing trials can be shared.

#### Epitope and target engagement

Target engagement is critical for drug discovery, and is determined by the specificity of the antibody, the ability to cross the BBB, or a combination thereof. A number of active and passive immunization studies reached clinical trial, but failed in phase III studies, possibly due to lack of target engagement. While these studies showed that systemic administration of Aβ1-42-targeting antibodies clear amyloid plaques, they do not prevent progressive cognitive decline in these patients (Holmes et al., 2008). DeMatthos et al. compared the murine IgG2b version of Bapineuzumab (3D6) at different stages of disease in experimental models (Demattos et al., 2012). The results showed that 3D6 is effective in preventing amyloid deposition in 9 month old PDAPP mice, while in 18–21 month aged PDAPP mice it fail to show any effect on amyloid load and showed an exacerbation of CAA-related microhemorrhage. The opposite effect was observed using an experimental antibody (mE8) that selectively recognizes a modified amino terminus of AβpE3-x, which effectively cleared Aβ by FcγR-mediated phagocytosis without vascular side effects. It was postulated that antibodies which bind both aggregated and soluble Aβ become saturated with soluble Aβ in the CNS and thus cannot engage the deposited Aβ, whereas the plaque-specific AβpE3-x antibodies robustly engages the deposited amyloid. These studies imply that antibody specificity may be most critical for successful development of Aβ immunotherapy. Plaque removal may not be sufficient to rescue AD memory decline and as a consequence, the concept of using antibodies targeting amyloid (Benilova et al., 2012) plaques has been regarded as a potential risk factor as plaques may serve as reservoirs of toxic Aβ peptides. Solanezumab, Crenezumab, Bapineuzumab, and Gantenerumab all bind to Aβ plaques in post-mortem and APP transgenic mouse tissue, albeit at different levels (Bouter et al., 2015; Fuller et al., 2015). Bapineuzumab does not recognize N-truncated or modified Aβ, while Solanezumab and Crenezumab do detect N-terminally modified Aβ peptides Aβ4-42 and pyroglutamate modified Aβ3-42 (Bouter et al., 2015). Immunotherapy using antibodies selective for Aβ4-42 and pyroglutamate-modified Aβ3-42, or the C-terminal modified Aβ1-37/38, without binding to plaques may be beneficial, although further understanding of the pathological relevance of truncated peptides is still required.

## Can antibody engineering improve efficacy?

**Figure 3:**
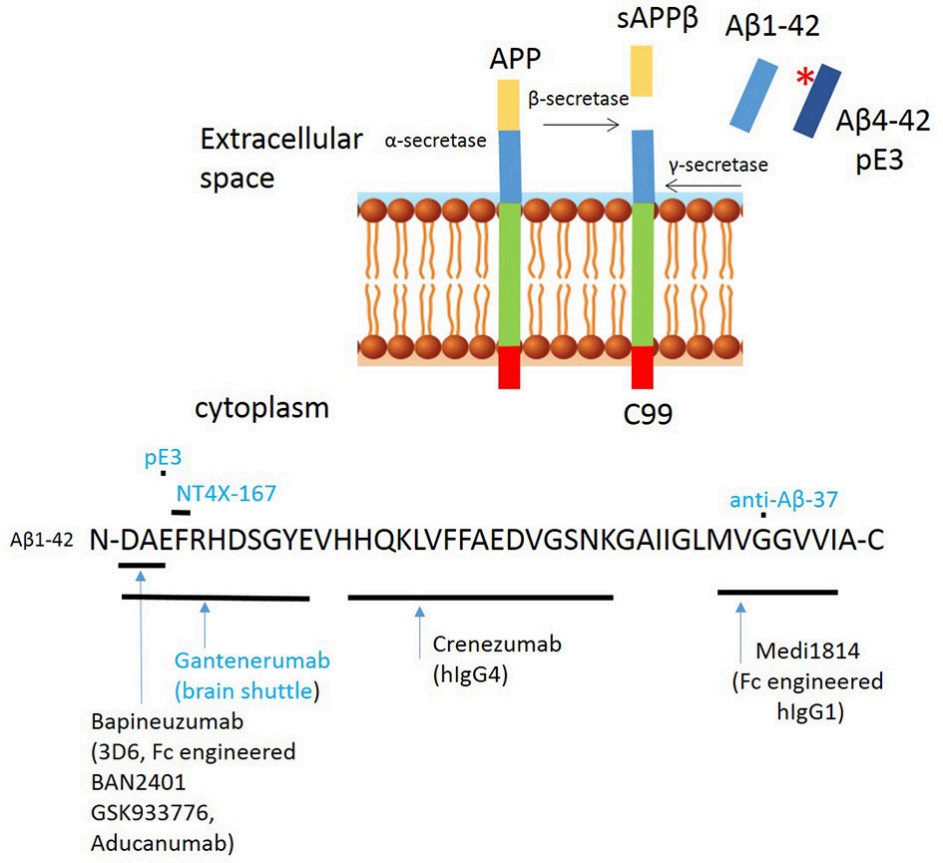
Proteolytic processing of amyloid precursor protein (APP) and binding sites of second generation anti amyloid β antibodies to Aβ peptide. Proteolytic processing of APP within the amyloidogenic pathway, including N-terminal modifications and binding sites of second/third generation antibodies targeting amyloid. Antibodies in blue have only been tested in experimental studies. ^*^Indicates pyroglutamination.

Several second generation antibodies are in development that address target engagement, including antibodies with highly specific epitopes, which are summarized in Table 1 and Figure 3). BAN2401 is a humanized IgG1 antibody that selectively binds to large soluble Aβ protofibrils (Lannfelt et al., 2014). Increased protofibril formation is found in a subgroup of AD patients, carrying the arctic APP mutation protein (E693G) (Nilsberth et al., 2001). This mutation leads to accelerated build-up of insoluble Aβ deposits both intraneuronal and/or extracellularly. BAN2401 was developed by the biotech company BioArctic Neuroscience, and licensed to Eisai and Biogen who are jointly developing this antibody for therapeutic use. A clinical study demonstrated that the incidence of ARIA-E on MRI was similar to placebo. It is currently in a Phase II trial in subjects with early AD (NCT01767311; Logovinsky et al., 2016). Another Aβ antibody, SAR255952 is a humanized monoclonal antibody also directed against soluble protofibrillar and fibrillar species of Aβ. This antibody is developed by Sanofi and is engineered on an IgG4 backbone which, like Crenezumab has low binding affinity for activating FcγRs on human microglia and no binding to complement C1q. A Phase 1 trial tested intravenous infusion and two doses of subcutaneous injection in patients with mild or moderate AD but no study results have been posted (NCT01485302). These studies highlight that stratification of patients carrying specific mutations in the APP gene may further contribute to the successful development of Aβ immunotherapy. Finally, the novel fully human monoclonal antibody Aducanumab, originally developed by the Swiss biopharmaceutical company Neurimmune and licensed by Biogen for clinical development, is revitalizing the “amyloid cascade hypothesis” (Weitz and Town, 2016).

**Table 1:** Overview of amyloid β specific antibodies.

**Antibody**	**Company**	**Current stage**	**Aβ type bound**	**Epitope**	**Subclass**	**ARIA-E Side effects**
Solanezumab	Eli Lilly	Phase 3 for mild AD—terminated	Circulating and soluble Aβ	Mid terminal. Residues 13-28	IgG1	No
Gantenerumab	Roche	Phase 3 for mild AD—ongoing	Fibrillary forms	Combined N terminus and mid-domain	IgG1	Yes
Crenezumab	Genentech/Roche	CREAD study phase 3 for mild AD—ongoing	Soluble oligomeric, fibrillary, and plaque	Mid terminus, residues 12–23	IgG4	No
Bapineuzumab	Elan/Pfizer and Johnson & Johnson	Phase 2 trial—terminated	Soluble and aggregated	N terminus. Residues 1–28	IgG1	Yes
Aducanumab	Biogen	Phase 3 ENGAGE—ongoing	Aggregated forms (insoluble fibrils and soluble oligomers)	N terminus	IgG1	Yes
Ponezumab	Pfizer Inc	Phase 3 trial for older individuals who may be at risk of memory loss-ongoing	Soluble and aggregated forms	Residues 33–40 in C terminus	IgG2a	No
BAN2401	Eisai/BioArctic Neuroscience	Phase 2b—ongoing	Soluble proto-fibrils	N terminal		No
SAR228810	Sanofi	Phase 1 completed	Pre-fibrillary aggregates		IgG4	No
GSK933776A	GSK	Phase 1 completed, no current plans to develop further		N terminal	IgG1	No

Aducanumab is a hIgG1 antibody and binds to the N-terminus of Aβ3-6. A unique feature of this antibody is its target: aggregated forms of the Aβ protein, both the insoluble fibrils and the soluble oligomers. This characteristic could circumvent the problem where soluble forms of Aβ may saturate antibodies such as Bapineuzumab meaning that plaques are not cleared effectively (Demattos et al., 2012). The antibody was originally discovered in elderly individuals with no signs of cognitive decline or cognitive decline with an unusually slow progression (Sevigny et al., 2016). Aducanumab was isolated by a process called “reverse translational medicine”^1^. This process starts by culturing B cells from the elderly population and screening for the ability of the culture supernatant to label Aβ plaques in brain tissue from APP transgenic mice and/or AD patients. Positive B cells, were further screened to remove antibodies with cross-reactivity with full length APP (Sevigny et al., 2016). Preclinical studies show penetration across the BBB and histological analysis of the brain showed that Aducanumab bound Aβ in both diffuse and compact plaques as well as Aβ associated with CAA. A IgG2a murine chimeric ^*ch*^Aducanumab was used to determine the efficacy to clear cerebral Aβ deposits. The results showed that Aβ40 and Aβ42 levels in the plasma brain were reduced in a dose-dependent manner. Thioflavin-S staining, which detect fibrillary amyloid, confirmed that Aducanumab reduced the number of plaques of all sizes and had no effect on vascular Aβ. Experiments further showed a significantly greater fraction of plaques that were at least 70% surrounded by microglia in the Aducanumab treated group than the PBS treated, suggesting FcγR mediated phagocytosis, which was confirmed by comparing plaque removal using an aglycosylated version of chAducanumab with a single N297Q point mutation to reduce FcγR binding (Sevigny et al., 2016). A study by Kastanenka et al. demonstrated how chronic administration of Aducanumab in 22 month old Tg2576 mice was able to ameliorate calcium overload and restore calcium homeostasis. Thus, this antibody may well-work by restoring the neuronal network function (Kastanenka et al., 2016). This study additionally shows that a readout of calcium overload may be a more appropriate readout for antibody efficacy than plaque reduction alone. Phase I studies followed to test the safety, tolerability and pharmacokinetics of Aducanumab (PRIME; ClinicalTrials.gov identifier NCT01677572) and no serious ARIA-E cases occurred in doses up 30 mg/kg. All patients receiving the 60 mg/kg dose developed ARIA-E and/or microhaemorrhage. The phase I study included PET imaging showing dose and time depend reduction of Aβ plaques, with the largest reduction in plaques in the 10 mg/kg^−1^ group after 1 year. Side effects were seen, most commonly Vasogenic Oedema, occurring early in treatment, but, none of these were serious and all were transient. Interestingly, analysis of the phase 1b study shown that a lowering of cerebral Aβ slows cognitive decline by both the Mini Mental State Examination (MMSE) & clinical Dementia rating. As a results of these studies Aducanumab received “Fast-Track Status” by the FDA and two phase 3 trials (ENGAGE & EMERGE) are ongoing to investigate the efficacy in slowing cognitive decline in patients with early AD. Results are expected for mid-2018. It is also very encouraging that, the extent of amyloid reduction with Aducanumab is more marked than observed in the previous trials of Gantenerumab and Bapineuzumab (Rinne et al., 2010; Ostrowitzki et al., 2012).

Drug delivery to the brain is a major roadblock to treatment of AD. Increased penetration can be achieved by antibody engineering and development of bispecific antibodies, for example targeting the transferring receptor expressed on the cerebral vasculature (Bien-Ly et al., 2014; Yu et al., 2014; Pardridge, 2016; Zuchero et al., 2016; see Table 2 for more details). A single chain Fv (ScFv) antibody form of an anti-amyloid antibody was fused to the CH3 domain of each heavy chain of a chimeric (mAb) against the mouse transferrin receptor (TfR); subcutaneous administration of this tetravalent bispecific antibody resulted in 60% reduction in amyloid (Sumbria et al., 2013). Roche recently developed a bispecific TfR-engineered version of Gantenerumab, called the brain shuttle (Niewoehner et al., 2014). Experimental studies revealed that the effector function of the bispecific antibody is camouflaged when the bispecific antibody is bound to TfR but fully active when it binds amyloid, its CNS target. It was postulated that this dual behavior is due to steric hindrance of FcγR on immune cells when TfR is bound. In this position, the two Fab arms of the IgG prevent the necessary proximity of the Fc region of the bispecific mAb to the FcγR on effector cells, possibly interfering with FcγR oligomerization. Once the bispecific mAb is released from the TfR into the CNS parenchyma, the FcγR on recruited microglia can be engaged, allowing efficient clearance. These experimental data provide a valid strategy for the use of fully effector-functional mAbs that can be transported safely across the BBB, but clinical validation is required.

**Table 2:** Overview of antibody engineering approaches.

**Alteration**	**More Info**	**Effect**	**Examples**
Bispecific antibodies	Antibodies targeting specific transport receptors at BBB	Increase penetration of therapeutic antibody to cross BBB	Anti-TfR/BACE1 Reduced Aβ in brain & CSF dose dependently (Yu et al., 2014). cTfRMAb-ScFv—anti Aβ + anti-TfR Increased transport over BBB, reduced Aβ load without increasing plasma Aβ and no CAA (Sumbria et al., 2013).
Glycosylation	E.g., addition of sialic acid	Reduced efflux from brain. No change on influx (Finke et al., 2017).	
Fc-engineering	Triple mutation in CH3	Reduced effector function—Fc Receptor and C1q binding	BAN AAB-003 MEDI

Alternative approaches may include modification of the glycosylation of therapeutic antibodies (Finke et al., 2017). Using *in vitro* models of brain microvascular endothelial cells, Banks et al. showed that mAb lacking sialic acid have reduced BBB penetrance compared to mAbs carrying sialic acid residues. The influx of antibodies appeared largely insensitive to changes in their glycosylation states. Thus, sialylation may offer a means to reduce IgG drug efflux from the brain in conjunction with other techniques and biochemical modifications that increase influx. Sialylation may also confer other clinical benefits to AD patients, as sialylated IgG is reported to induce immune pathways with minimal inflammation (Li et al., 2017).

## Immunotherapy beyond amyloid

The development of second generation anti-amyloid antibodies are showing renewed interest in this therapeutic approach based on promising effects in clinical trials, but it is important to keep in mind the diverse underlying biological mechanism of AD, including the role of established genetic and environmental risk factors. At least four disease mechanisms have been implicated in AD besides aggregation of beta amyloid and tau: neuroinflammation, vascular pathology, loss of protein homeostasis, and mitochondrial dysfunction. Immunotherapies targeting proteins other than amyloid are in clinicial development, including anti-tau mAbs. For example, AbbVie 8E12 recognizes extracellular, aggregated tau, and Genentech humanized RO7105705, a mouse monoclonal antibody, which targets tau in the extracellular space, hoping to block the spread of toxic forms (Pedersen and Sigurdsson, 2015; Lee et al., 2016). Other approaches include peripheral administration of ApoE antibodies using experimental models, resulting in reduced ApoE levels in the brain, improved spatial learning performance in the Morris water maze and enhanced resting-state bilateral functional connectivity in different cortical regions. *In vivo* two-photon microscopic imaging demonstrated that ApoE specific antibodies are capable of reducing amyloid load. ApoE carriers have higher amyloid load in the parenchyma and cerebral vasculature and increased risk of cardiovascular disease and inflammation. It is now well-accepted that inflammation has a key role in sporadic AD and it is hypothesized that the APOE-ε4 allele, modulates inflammation in both the periphery and the brain (Whalley et al., 2006; Harold et al., 2009; Hollingworth et al., 2011; Benitez et al., 2014).

Population studies have provided good evidence that a high cardiovascular risk profile reliably predicts progression from mild cognitive impairment to AD (Viticchi et al., 2015, 2017). Indeed, vascular cognitive impairment and dementia (VCID, or vascular dementia), occurs in as much as 40% of AD patients (Zekry et al., 2002), which may not be routinely taken into account in clinical trial design. An interesting experimental model for vascular dementia is hyperhomocysteinemia (HHcy), which can be induced by a diet deficient in folate, B6, and B12 and supplemented with excess methionine inducing diet (Sudduth et al., 2013). Elevated circulating levels of the sulfur-containing amino acid homocysteine can lead to oxidative stress, pro-inflammatory cytokine production, increased levels of inducible nitric oxide (NO) synthase (iNOS), and cerebral vascular dysfunction (Faraci and Lentz, 2004; Kamat et al., 2015). Weekman et al. tested the efficacy of Aβ immunotherapy in APP/PS1 mice fed the HHcy or control diet (Weekman et al., 2016). Intriguingly, systemic 3D6 (IgG2a) treatment failed to improve cognition in APP/PS1 mice on the HHcy diet, despite reduced levels of total Aβ levels. This lack of cognitive benefits could be due to the increase in the number of microhemorrhages seen in the 3D6-treated HHcy mice compared with the 3D6 or HHcy controls, or the increased levels of CAA. The lack of cognitive effects was not associated with an overt cytokine response by microglia, suggesting alternative underlying mechanism. These finding could be of importance as VCID is asymptomatic and may be a common co-morbidity in mild AD. AD pathology is characterized by the presence of Aβ oligomers and fibers, which are typically found in the aging brain. There are numerous endogenous proteins and metal ions that interact with Aβ and influence its assembly process both *in vitro* and in animal models. One of these proteins is albumin, which prevents fibrillization upon binding (Bode et al., 2018). The ability of albumin to sequester Aβ may explain why Aβ deposits are not observed in the peripheral vasculature, even though plasma levels of Aß are comparable to CSF (Bode et al., 2018). Albumin plays a role in clearing amyloid from the brain and the use of albumin as a therapeutic approach has shown encouraging effects in clinical trial (Boada et al., 2017). Interestingly, cholesterol and fatty acids prevent albumin from binding to amyloid, possibly further explaining cardiovascular disease as a link to AD (Bode et al., 2018).

Systemic inflammation contributes to an altered CNS microenvironment, switching primed microglial into a more aggressive phenotype, changes to the BBB permeability and increased expression of FcγRs on microglia and perivascular macrophages (Teeling and Perry, 2009; Lunnon et al., 2011). Tucsek et al. made similar observations when comparing the effect of a high fat diet showed increased BBB leakage and levels of IgG in the hippocampus of aged mice, but not young mice (Tucsek et al., 2014). These changes were accompanied by increased transcriptional levels of pro-inflammatory cytokines. Collectively, these experimental studies imply that that low grade systemic inflammation, induced by diet or chronic bacterial infection (Ide et al., 2016), alters the microenvironment of the brain and thereby, possibly the efficacy of amyloid immunotherapy; biomarkers of systemic inflammation may be a useful addition to stratify patients in future clinical trials.

## Conclusion

The clinical trials to date have shown that immunotherapy against Aβ is able to clear plaques from the brains of AD patients. However, they have also highlighted the danger of immune activation within the CNS, as a result of neuroinflammation and vasogenic oedema. Lessons can be learnt from cancer immunotherapy where monoclonal antibodies have been engineered to improve efficacy and allowing higher antibody penetration with fewer side effects. In addition, antibody specificity appears critical as shown in recent data using the fully human antibody Aducanumab, which selectively binds to the aggregated form of amyloid and encouraging results in preclinical models using antibodies that selectively bind to truncated amyloid peptides. To allow the production of safe and effective CNS immunotherapies it is essential to understand the underlying biological mechanisms that can contribute to antibody efficacy, which may include genetics and environmental factors. This would allow the selection the most appropriate antibody isotypes or mutants minimizing the risk of adverse events.

## Author contributions

IS: contributed to writing the manuscript; RE: contributed to writing the manuscript; AA: contributed to writing and reviewing the manuscript; JT: contributed to writing and reviewing the manuscript.

### Conflict of interest statement

AA is a full time employee of H lundbeck A/S. JT and RE have received funding from H Lundbeck A/S. The other author declares that the research was conducted in the absence of any commercial or financial relationships that could be construed as a potential conflict of interest.

## Footnotes

^1^Biogen (2016). www.biogen.com.
